# Are Microglial Cells the Regulators of Lymphocyte Responses in the CNS?

**DOI:** 10.3389/fncel.2015.00440

**Published:** 2015-11-16

**Authors:** Beatriz Almolda, Berta González, Bernardo Castellano

**Affiliations:** Department of Cell Biology, Physiology and Immunology, Facultat de Medicina, Institute of Neurosciences, Universitat Autònoma de BarcelonaBellaterra, Spain

**Keywords:** antigen presentation, lymphocyte, dendritic cells, co-stimulatory signals, MHCs, B7, purine nucleotides, CD39

## Abstract

The infiltration of immune cells in the central nervous system is a common hallmark in different neuroinflammatory conditions. Accumulating evidence indicates that resident glial cells can establish a cross-talk with infiltrated immune cells, including T-cells, regulating their recruitment, activation and function within the CNS. Although the healthy CNS has been thought to be devoid of professional dendritic cells (DCs), numerous studies have reported the presence of a population of DCs in specific locations such as the meninges, choroid plexuses and the perivascular space. Moreover, the infiltration of DC precursors during neuroinflammatory situations has been proposed, suggesting a putative role of these cells in the regulation of lymphocyte activity within the CNS. On the other hand, under specific circumstances, microglial cells are able to acquire a phenotype of DC expressing a wide range of molecules that equip these cells with all the necessary machinery for communication with T-cells. In this review, we summarize the current knowledge on the expression of molecules involved in the cross-talk with T-cells in both microglial cells and DCs and discuss the potential contribution of each of these cell populations on the control of lymphocyte function within the CNS.

## Introduction

The central nervous system (CNS) has been considered for many years as an organ immunologically isolated from the peripheral immune system, on one hand due to the presence of the blood brain barrier (BBB) and the absence of lymphatic vessels (Perry, 1998) and, on the other hand, by the fact that skin grafts and the direct inoculation of viruses, bacteria or antigens in the nervous parenchyma did not induce an immune response (Medawar, 1948; Barker and Billingham, 1977; Stevenson et al., 1997; Matyszak and Perry, 1998). Nevertheless, in the last decade, an increasing number of studies has demonstrated that the CNS is not only immune-competent, but it also actively interacts with cells of the peripheral immune system (Aloisi et al., 2000; Becher et al., 2000; Steinman, 2004; Almolda et al., 2011b; Gonzalez et al., 2014), which can be recruited to the nervous parenchyma under specific circumstances (Ransohoff et al., 2003; Engelhardt and Ransohoff, 2005; Becher et al., 2006; Engelhardt, 2006, 2008).

With all of these studies in mind, it is easy to think that the isolated view of the CNS has drastically changed toward a more active scenario, in which a situation of active immune tolerance is continuously maintained within the CNS. Different mechanisms have been reported to contribute to this active tolerance, including the constitutive expression of FasL, a receptor involved in the death of infiltrated immune cells (Bechmann et al., 1999; Flugel et al., 2000) and the local production of anti-inflammatory mediators such as indolamine 2,3-dioxygenase, in response to the interaction with pro-inflammatory lymphocytes (Kwidzinski et al., 2005). The presence of some populations of cells, such as macrophages and dendritic cells (DCs), located in strategic areas of the CNS such as the meninges and the choroid plexus, may play a key function in the initiation and regulation of immune responses. Nowadays, then, the CNS is considered as an immune-privileged site, rather than immune-isolated (Ousman and Kubes, 2012; Ransohoff and Engelhardt, 2012).

## Infiltration of Lymphocytes in the CNS Under Pathological Situations

The infiltration of lymphocytes within the CNS parenchyma is a common hallmark in many pathological conditions (Rezai-Zadeh et al., 2009; Anderson et al., 2014) such as VIH (Petito et al., 2003) and West Nile virus infection (Glass et al., 2005); neurodegenerative diseases such as Parkinson’s disease (Brochard et al., 2009) and amyotrophic lateral sclerosis (Holmoy, 2008); acute lesions like facial nerve axotomy (Raivich et al., 1998), entorhinal cortex lesion (Babcock et al., 2008), stroke (Schroeter et al., 1994; Gelderblom et al., 2009) and ischemia (Gelderblom et al., 2009) or autoimmune processes such as experimental autoimmune encephalomyelitis (Dittel, 2008; Almolda et al., 2011a). While in some circumstances lymphocyte infiltration has been related to protective functions, as occurs in the facial nerve axotomy paradigm (Serpe et al., 1999), the West Nile virus infection (Glass et al., 2005) and amyotrophic lateral sclerosis (Beers et al., 2008; Chiu et al., 2008), in other circumstances lymphocyte infiltration has been shown to contribute to the exacerbation of the pathology. This is the case of Parkinson’s disease (Brochard et al., 2009), VIH virus infection (Petito et al., 2003), stroke (Yilmaz et al., 2006) and some autoimmune diseases (Dittel, 2008).

Due to the fact that T-cells are not able to recognize soluble antigens, they need the help of specialized cells, the so-called antigen presenting cells (APCs), which through antigen presentation mechanisms can capture, process and present pathogen and viral antigens and other strange structures for recognition by T-cells. Depending on the pattern of cytokine secretion, the functions and the molecules that drive their differentiation, different subtypes of T-helper lymphocytes are identified (Reinhardt et al., 2006; Takatori et al., 2008; Sun and Zhang, 2014). Classical classification considers two different subtypes: T-helper 1 (Th1) lymphocytes, which secrete pro-inflammatory cytokines such as interferon-γ (IFN-γ) or tumoral necrosis factor-α (TNF-α) and Th2 lymphocytes, which produce anti-inflammatory cytokines such as interleukin-4 (IL-4) and interleukin-10 (IL-10). Therefore, Th1 accumulation has been usually considered as an inflammatory event, whereas presence of Th2 has been related to the down-regulation of the inflammatory response. However, a growing accumulation of evidence has changed this simple paradigm based on the presence/absence of Th1/Th2, as other subpopulations of Th cells have been discovered, among them, effector T-cells including Th17, Th22, Th9, T-follicular helper (Tfh) cells with the capacity to secrete different cytokines (Cosmi et al., 2014), but also regulatory T-cells such as T-regulatory (Treg) and Tr1, whose principal function is to maintain the immune system homeostasis and the tolerance to self-antigens (Bluestone and Tang, 2005; Eltzschig et al., 2012; Piccioni et al., 2014). Two different subtypes of Treg are currently identified: the natural Treg (nTreg) and the induced Treg (iTreg) (Horwitz et al., 2008; Curotto de Lafaille and Lafaille, 2009; Piccioni et al., 2014). The nTregs, defined as CD4+CD25+Foxp3+ cells, are generated in the thymus during the maturation of T-cells by recognition of self-peptides with intermediate affinity, whereas the iTregs are produced in secondary lymphoid organs (spleen and lymph nodes) from naïve CD4+Foxp3- T-cells under both homeostatic conditions and in the presence of inflammation, infection or allergy after stimulation with TGF-β (Piccioni et al., 2014). Due to their capacity to suppress immune responses, the participation of Tregs in the evolution of acquired immune responses in the CNS, especially those related to autoimmunity, has generated much attention in the last several years. In this sense a remarkable accumulation of Tregs in cerebral gliomas (Grauer et al., 2007), ischemic stroke (Stubbe et al., 2012) and in some experimental models of encephalomyelitis such as EAE (McGeachy et al., 2005; Kohm et al., 2006; Korn et al., 2007) has been reported.

The discovery of all of these subtypes of lymphocytes with putative new functions in the promotion and modulation of the acquired immune response and their still-unknown interactions with resident CNS cells, specially microglia, has contributed to becoming aware that the scenario of the neuroimmune response could be even more complicated than previously thought.

## Activated Microglia are Considered the Main APC in the CNS

Microglial cells are considered the sole representative of the immune system within the CNS parenchyma. The precise origin of microglia during development still remains under debate, although emerging evidence reported that yolk-salk primitive precursors are the principal source (Ginhoux et al., 2010, 2013; Schulz et al., 2012). Studies in bone-marrow chimera and parabiotic mice indicated that these yolk-salk precursors invade the CNS parenchyma through the blood vessels around embryonic Day 9 in mice, corresponding to the vascularization process, and contribute substantially to the maintenance of microglial cells in the adult (Ginhoux et al., 2010). However, alternative routes of entry for microglial precursors, including the ventricles and meninges, have been identified (Cuadros and Navascues, 1998; Dalmau et al., 1998, 2003; Navascues et al., 2000). Whether these different routes of entry are linked to different populations of microglial precursors with different functions is an interesting field that is still unsolved.

Microglial cells are equipped with a broad range of receptors in their plasma membrane that allows them to sense subtle changes in the micro-environment (Kettenmann et al., 2011; Hanisch, 2013; Kierdorf and Prinz, 2013). Microglial cells play very important roles in healthy, normal CNS, not only during the post-natal period, where they contribute to the elimination of synaptic structures (Pont-Lezica et al., 2011; Tremblay et al., 2011; Harry and Kraft, 2012), but also in the adult, where they are continuously scanning their local micro-environment (Davalos et al., 2005; Nimmerjahn et al., 2005; Kierdorf and Prinz, 2013; Castellano et al., 2015). When the homeostasis of the CNS is perturbed as a result of injury or disease, microglial cells become rapidly activated, acquiring a specific phenotype totally dependent on the environment in which they are activated and the specific stimulus that drives their activation (Kettenmann et al., 2011; Gonzalez et al., 2014; Chen and Trapp, 2015). Activated microglia can rapidly proliferate and increase the expression or *de novo* express a multitude of different molecules and secrete a plethora of substances such as cytokines, chemokines and trophic factors, all of which make them able to modulate both the innate and the acquired immune responses within the CNS (Ransohoff and Cardona, 2010; Kettenmann et al., 2011; Eggen et al., 2013; Goldmann and Prinz, 2013; Casano and Peri, 2015).

Recognition of the T-cell receptor (TCR) on the surface of T-lymphocytes by the major histocompatibility complexes (MHCs) located on the surface of the APCs, MHC-I in the case of CD8+T-cytotoxic lymphocytes and MHC-II for CD4+T-helper cells, constitutes the first signal of the antigen-presenting mechanism related to the activation of T-cells (Lanzavecchia, 1997; Abbas et al., 2010). Co-stimulation, the second signal involved in this mechanism, is based on the binding of diverse receptors and counter-receptors expressed on the surface of both APC and T-cells (Nurieva et al., 2009) and is essential for a complete antigen presentation, as expression of MHCs in the absence of co-stimulation leads to the apoptosis or anergy of T-cells (Kishimoto and Sprent, 1999). A multitude of co-stimulatory pairs of molecules, which can be classified into two main families (the B7/CD28 and the TNFR families), have been reported in the immune system, exerting different effects on the activation/deactivation of T-cells (Sharpe, 2009) and driving the final outcome and function of T-cells.

### Expression of MHCs in Microglia

Resident glial cells, principally microglia, can establish a cross-talk with infiltrated T-cells regulating their recruitment, activation and function within the CNS (Gonzalez et al., 2014). Although in healthy CNS microglial cells do not express MHCs (Kreutzberg, 1996; Perry, 1998), it is well known that, when activated in pathological conditions, they showed a wide number of phenotypic changes (Ransohoff and Cardona, 2010; Kettenmann et al., 2011; Prinz et al., 2014), including *de novo* expression of these molecules (Kreutzberg, 1996; Perry, 1998). Therefore, many authors consider microglial cells as the principal APC within the CNS parenchyma (Aloisi, 2001; Carson, 2002; Raivich and Banati, 2004; Graeber and Streit, 2010). Expression of MHC-II in activated microglia *in vivo* has been reported after a wide variety of CNS injuries including LPS injection (Xu and Ling, 1995; Ng and Ling, 1997), ischemia and kainic acid injection (Finsen et al., 1993), graft *vs.* host disease (Sedgwick et al., 1998), facial nerve axotomy (Streit et al., 1989; Villacampa et al., 2015), entorhinal cortex lesion (Bechmann et al., 2001; Kwidzinski et al., 2003a) and different models of EAE (Almolda et al., 2010).

### Expression of Co-stimulatory Molecules in Microglia

While the expression of MHCs has been extensively reported in activated microglia, only a limited number of studies have addressed the question of whether activated MHC-II+ microglia simultaneously express co-stimulatory molecules (Summarized in **Table 1**).

**Table 1:** Principal co-stimulatory molecules from the B7/CD28 and TNFR family.

	Effect on T-cell	T-cell	APC	Determined in microglia	Experimental model	Reference
B7/CD28 family					PPT	Bechmann et al., 2001 Kwidzinski et al., 2003b
	Stimulation	CD28				
					Peripheral nerve injury	Rutkowski et al., 2004
	
					FNA	Bohatschek et al., 2004
				
			B7.1/B7.2	+	Cuprizone	Remington et al., 2007
	
	Inhibition	CTLA-4			EAE models	Issazadeh et al., 1998 Juedes and Ruddle, 2001 Mack et al., 2003 Raivich and Banati, 2004 Almolda et al., 2010 Almolda et al., 2011b
	
	Stimulation	ICOS	B7H2 (ICOS-L)	n.d.	–	–
	
					MCAO	Ren et al., 2011 Bodhankar et al., 2013
	
					Coronavirus infection	Phares et al., 2009
	
					TMEV	Duncan and Miller, 2011 Jin et al., 2013
	Inhibition	PD-1	B7H1 (PD-L1)	+		
					EAE models	Schreiner et al., 2008
	
					PPT	Lipp et al., 2007
	
			B7DC (PD-L2)	n.d.	–	–
	
	Inhibition	TLT-2	B7H3	n.d.	–	–
	
	Inhibition	Unknown	B7H4	n.d.	–	–
	
	Inhibition	Unknown	B7S3	n.d.	–	–
	
	Inhibition	Unknown	BTNL	n.d.	–	–

TNFR family					Microglial cultures	Tan et al., 1999 Qin et al., 2005 Lin et al., 2009 Lin and Levison, 2009 Vidyadaran et al., 2009
	
					Ageing	Griffin et al., 2006 Simpson et al., 2007
	
					Epilepsy	Sun et al., 2008
	
					Alzheimer’s disease	Togo et al., 2000 Town et al., 2001 Tan et al., 2002a
	Stimulation	CD40-L	CD40	+		
					ALS	Okuno et al., 2004
	
					Neurodegeneration	Ke et al., 2005
	
					HIV infection	D’Aversa et al., 2002, 2005
	
					EAE models	Becher et al., 2001; Ponomarev et al., 2006
	
					TMEV	Olson et al., 2001
	
					MS	Vogel et al., 2013
	
	Stimulation	OX40	OX40-L	n.d.	–	–
	
	Stimulation	CD27	CD70	n.d.	–	–

*The table summarized the different molecules studied in the context of microglial cells (+). n.d indicates that the expression has not been determined specifically in microglia. PPT, perforant pathway transection; FNA, facial nerve axotomy; EAE, experimental autoimmune encephalomyelitis; MCAO, middle cerebral artery occlusion; TMEV, Theiler’s induced encephalitis; ALS, amyotrophic lateral sclerosis; MS, multiple sclerosis.*

#### The B7/CD28 Family

The pair of co-stimulatory molecules with the major relevance in the activation of T-cells, and therefore the most extensively studied in the organism, is that formed by receptors B7.1/B7.2 (CD80/CD86) on the surface of APCs and their counter-receptors CD28 and CTLA-4 on the surface of T-cells. The binding of B7.1 or B7.2 to CD28 induces T-cell proliferation and cytokine secretion, whereas binding of these same receptors to CTLA-4 induces the inhibition of T-cell activity, promoting the down-regulation of the immune response (Sansom, 2000; Sharpe and Freeman, 2002). Specifically in the CNS, *de novo* expression of B7.1 and/or B7.2 has been reported in microglial cells after entorhinal cortex lesion (Bechmann et al., 2001; Kwidzinski et al., 2003b), peripheral nerve injury (Rutkowski et al., 2004), facial nerve axotomy (Bohatschek et al., 2004), cuprizone-induced demyelination (Remington et al., 2007) and models of autoimmunity such as EAE and Theiler’s virus encephalomyelitis (Issazadeh et al., 1998; Juedes and Ruddle, 2001; Mack et al., 2003; Raivich and Banati, 2004; Almolda et al., 2010, 2011b).

Recently, other members of the B7 co-stimulatory molecules family have been described in the immune system, including B7-H2 (ICOS-L), B7-H1 (PD-L1), B7-DC (PD-L2), B7H3 (CD276), B7H4, B7S3 and BTNL (Sharpe, 2009; Chen and Flies, 2013). The ICOS-ICOSL pathway has important roles in the fine-tuning of effector T-cell functions and the control of T-cell tolerance (Nurieva et al., 2009). Although the presence of ICOS+ T-cells has been reported in the CNS of EAE-induced mice (Rottman et al., 2001), to-date, no studies on the expression of its ICOSL ligand on microglia or any other CNS resident cells are available. PD-1 is another receptor gaining attention, due to its crucial role in maintaining peripheral immune tolerance (Nurieva et al., 2009). PD-1 has been shown to be a negative regulator of T-cell responses, expressed at low levels on the surface of T, B and natural killer T-cells, and further induced upon activation. PD-1 has two counter-receptors that are expressed on the surface of APCs, PD-L1 and PD-L2 also called B7H1 and B7DC, respectively (Nurieva et al., 2009). The few reports addressing the expression of this molecule in the CNS demonstrated PD-L1 expression in both activated microglia after middle-cerebral artery occlusion (Ren et al., 2011; Bodhankar et al., 2013), coronavirus infection (Phares et al., 2009), Theiler’s murine encephalomyelitis (Duncan and Miller, 2011; Jin et al., 2013) and EAE (Schreiner et al., 2008), and in astrocytes after entorhinal cortex lesion (Lipp et al., 2007). Moreover, the blockade of PD-1 signaling enhances EAE severity (Salama et al., 2003) suggesting an outstanding role in the control of CNS pathologies.

To our knowledge, no studies regarding the expression of B7H3, B7H4, B7S3 or BTNL specifically in microglia are, until present, available in the literature.

#### The TNFR Family

Additionally, a second family of co-stimulatory receptors, the TNFR family, has been reported in the immune system. Various members, including pairs CD40/CD40L, OX40L/OX40, and CD70/CD27, expressed on APCs and T-cells, respectively, form this family (Watts, 2005; Sharpe, 2009). Among them, CD40 is the only molecule studied in the context of microglial activation (Chen et al., 2006). CD40 expression in activated microglia has been described not only *in vitro* in many cell-lines activated with IFN-γ, LPS or β-amyloid protein (Tan et al., 1999; Qin et al., 2005; Lin and Levison, 2009; Lin et al., 2009; Vidyadaran et al., 2009) but also *in vivo* during physiological aging (Griffin et al., 2006; Simpson et al., 2007) and under pathological situations such as epilepsy (Sun et al., 2008), Alzheimer’s disease (Togo et al., 2000; Town et al., 2001; Tan et al., 2002b), amyotrophic lateral sclerosis (Okuno et al., 2004), neurodegeneration induced by thiamine deficiency (Ke et al., 2005), human HIV (D’Aversa et al., 2002, 2005), different animal models of autoimmunity such as EAE (Becher et al., 2001; Ponomarev et al., 2006) and Theiler’s murine encephalomyelitis (Olson et al., 2001) and MS (Vogel et al., 2013). Moreover, inhibition of CD40 in microglia results in the attenuation of β-amyloid pathology (Tan et al., 2002a) and the reduction of EAE severity (Becher et al., 2001; Ponomarev et al., 2006), pointing toward this molecule as a good candidate for therapeutic interventions in these specific CNS pathologies.

Altogether, these studies indicate that, although so far it seems that microglial cells may be the principal APC within the CNS, in the coming years it will be necessary to inquire about the expression of some other markers related to the antigen-presenting mechanism described in professional DCs and, until now, not explored in the context of microglial activation.

## Other Molecules Expressed by Microglia that can be Involved in the Communication with T-Cells

Recent studies indicate that CD39 and CD73, some of the molecules that mediate the immunosuppressive activity of Treg lymphocytes (Deaglio et al., 2007), are also expressed in specific subtypes of APCs and may be involved in the suppressive activity of these cells. Specifically, a subtype of DCs induced by IL27 has been shown to increase expression of CD39 and exert protective functions in EAE (Mascanfroni et al., 2013). CD39 and CD73 (also known as NDPase and 5′ nucleotidase, respectively) are enzymes involved in the hydrolysis of extracellular ATP to ADP/AMP and to adenosine. CD39-deficiency in DCs has been shown to ameliorate the course of EAE by reducing the number of Th1 and Th17 effector cells (Mascanfroni et al., 2013). The precise mechanism by which CD39 regulates T-cell responses is not clear, although it is proposed to be mediated by a reduction in the ATP levels producing a down-regulation of the inflammasome activity (Eltzschig et al., 2012), a multiprotein-assembled complex involved in the initiation of the immune innate responses (Vanaja et al., 2015).

Expression of both CD39 and CD73 in the membrane of microglial cells has been extensively reported to regulate ATP levels within the CNS, in both healthy situations and after damage (Castellano et al., 2015). Therefore, it is easy to suggest that regulation of the expression of those enzymes in activated microglia take part in modulating the final outcome of infiltrated T-cells.

## Presence of Dendritic Cells in the CNS

Dendritic cells are considered to be the professional APCs in the immune system (Guermonprez et al., 2002). They are derived from hematopoietic stem cells in the bone marrow that gives rise to early precursors called the Common Myeloid Precursor (CMP). CMPs, in turn, originate the formation of two different precursors, the Granulocyte/Monocyte precursors (G/Ms) and the Macrophage/DC precursors (M/DPs). From M/DPs, the common DC progenitors, the pre-DC precursors and the plasmacytoid DCs are sequentially formed. Pre-DC precursors egress into the blood circulation and populate different organs, including the skin, heart, lung and spleen, becoming conventional DCs (Liu and Nussenzweig, 2010). As both DCs and macrophages derived from the same precursors most of the markers and functions of these two populations are similar.

Although the parenchyma of the normal CNS are devoid of the so-called professional DCs, these cells are abundant in the meninges, the choroid plexus (McMenamin, 1999; McMenamin et al., 2003), the perivascular space and the juxtavascular parenchyma (i.e., the neuropil just beyond the glia limitants) (Prodinger et al., 2010). These locations are considered strategically well-positioned for the communication with blood-circulating pathogens or T-cells, supporting a role of DCs in the control of the entry gates to the brain and thus in the regulation of immune surveillance in the CNS during homeostasis. With aging, the number of DCs increases markedly in the perivascular space, meninges and choroid plexuses, and has even been found into the brain parenchyma (Stichel and Luebbert, 2007; Kaunzner et al., 2010). The presence of CNS parenchymal DCs has also been reported in different neuroinflammatory situations (McMahon et al., 2006; Colton, 2012; D’Agostino et al., 2012), including infections (Fischer and Reichmann, 2001), traumatic brain injury (Israelsson et al., 2010), ischemia (Kostulas et al., 2002; Reichmann et al., 2002; Felger et al., 2009; Gelderblom et al., 2009), excitotoxicity (Newman et al., 2005) and some diseases such as amyotrophic lateral sclerosis (Henkel et al., 2004), multiple sclerosis (Plumb et al., 2003; Serafini et al., 2006) and EAE (Matyszak and Perry, 1996; Serafini et al., 2000; Fischer and Reichmann, 2001; Santambrogio et al., 2001; Santambrogio and Strominger, 2006; Almolda et al., 2010, 2011b).

## Function of DCS in the CNS

Numerous works (McMahon et al., 2006; Colton, 2012; D’Agostino et al., 2012) emphasize the possible relevance of DCs in the CNS immunosurveillance as well as the function they can play in neuroinflammatory situations. However, the specific contribution of those cells is still not well-understood.

The actual knowledge regarding the function of DCs in the brain come from studies using the inoculation of different types of DCs into the CNS under different circumstances. Thus, it has been shown that subcutaneous administration of bone marrow DCs before EAE-induction prevents EAE development in rats (Huang et al., 2000). Other studies reported that intraparenchymal inoculation of tolerogenic DCs, induced by TNF-α treatment, prevents or delays EAE onset, whereas immunogenic DCs administration increases the severity of this disease (Zozulya et al., 2009).

All together, these studies have demonstrated the potential of DCs to serve as potent vehicles to induce tolerance and open a door to new therapeutic strategies to modulate CNS disease. A question not yet addressed in this kind of studies is how these DCs interact with both glial cells and blood–borne infiltrated cells. Research in this field in the coming years is vital to understand the molecular and cellular mechanisms involved in the regulation of immune responses in the CNS.

## Are CNS Parenchymal DCS Authentic DCS or are they a Subtype of Activated Microglia?

**FIGURE 1:**
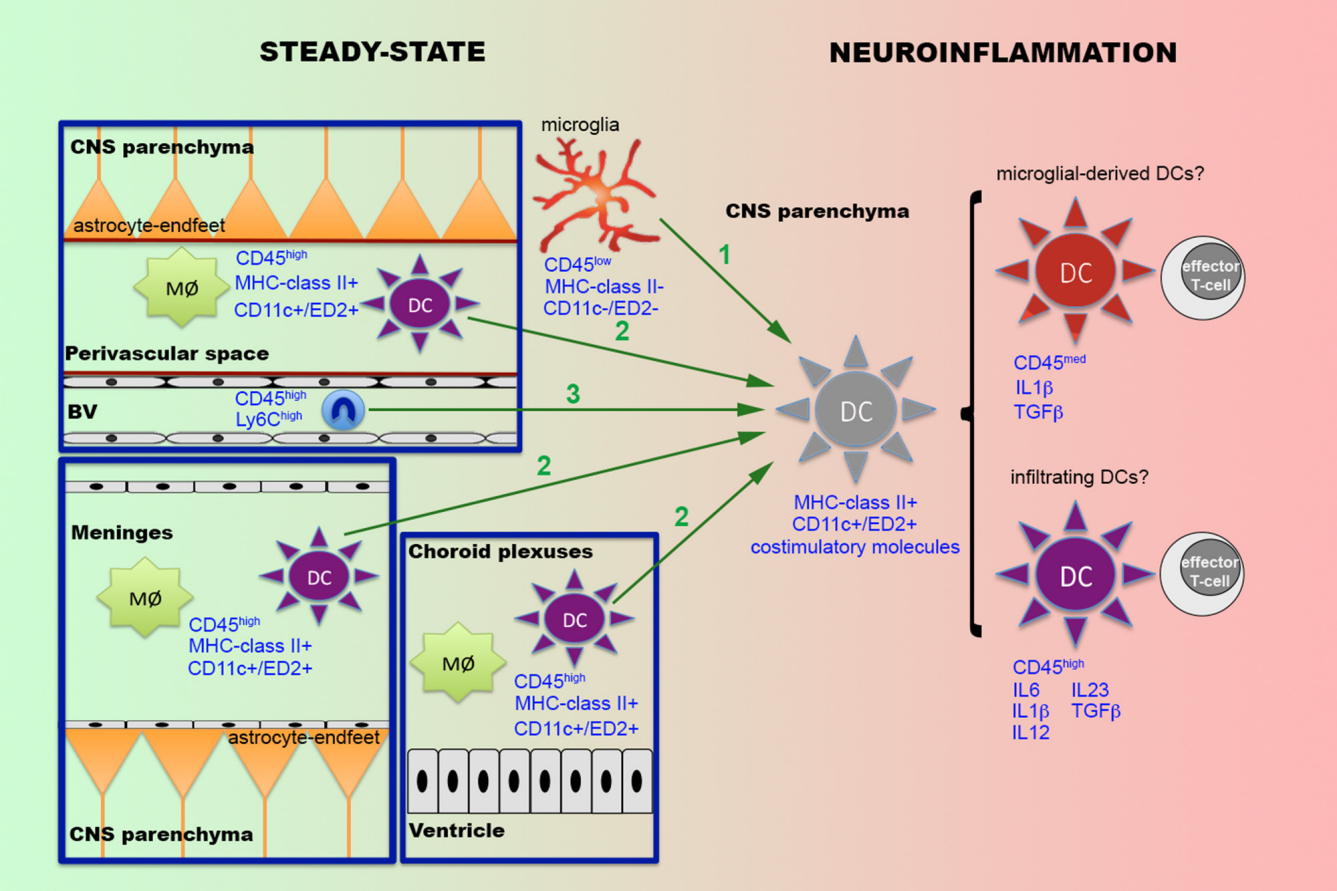
**Putative origins of parenchymal dendritic cells during neuroinflammatory conditions.** In the CNS, under steady-state conditions a population of professional DCs expressing MHC-class II and CD11c or ED2 are found in the meninges, the choroid plexuses and the perivascular space, where they coexist with specific subpopulations of resident macrophages (Mø). Under specific neuroinflammatory conditions, such as infections, traumatic brain injury or EAE, DCs have been also reported within the CNS parenchyma. Different possibilities are suggested to explain the origin of these parenchymal DCs (green arrows). The first possibility (1) is that parenchymal DCs are derived from activated microglia. The second possibility (2) is that parenchymal DCs come from the recruitment of either perivascular or meningeal or both DC populations. The third possibility (3) is that DCs come from infiltrated monocytes (Ly6C^high^). Recent research indicates that parenchymal DCs are constituted by two different populations of cells, one becoming from microglia and the other infiltrated from the periphery. Although both populations of parenchymal DCs present the ability to activate T-cells, the fact that they display a distinct phenotype, characterized principally by changes in the levels of CD45 and the pattern of cytokine secretion, suggest that they may play different roles in the regulation of the immune response.

In addition to the poor knowledge on the role of DCs in the immune responses within the CNS, one of the issues that generate more controversy is the origin of parenchymal DCs observed in a wide range of neuroinflammatory situations (**Figure 1**). One possibility suggested by some authors is that the perivascular or meningeal DCs observed in the healthy brain are recruited to inflammatory sites within the CNS parenchyma (McMahon et al., 2006). Alternatively, other authors supported the idea that parenchymal DCs observed during neuroinflammatory conditions come from infiltrated monocytes (Ifergan et al., 2008), which under the influence of specific molecules such as GM-CSF, differentiate to DCs (Ashhurst et al., 2014). Supporting this idea, an alternative developmental circuit occurring after the MDP precursors involves monocytes as precursors of inflammatory DCs in peripheral organs (Dominguez and Ardavin, 2010; Liu and Nussenzweig, 2010). Infiltration of monocytes is a common event in many of the above-mentioned neuroinflammatory situations in which DCs have been described in the CNS parenchyma (Zhu et al., 2007; Serbina et al., 2008; Mildner et al., 2009). Furthermore, systemic administration of GM-CSF in EAE-induced mice mobilizes Ly6C^high^-circulating monocytes that migrate to the CNS parenchyma and are converted into DCs (King et al., 2009). Nevertheless, later studies have demonstrated that intraparenchymal infusion of GM-CSF not only promotes the apparition of DC precursors recruited from the periphery but also induces the emergence of a second population of DCs derived from the CNS with an inhibitory phenotype (Hesske et al., 2010), supporting the idea that DCs not only immigrate from the periphery but may also be derived from local CNS cells.

In this regard, several lines of evidence, including *in vitro* studies (Fischer and Reichmann, 2001; Butovsky et al., 2007) and neuroinflammatory situations such as ischemia (Kostulas et al., 2002) and EAE (Fischer and Reichmann, 2001; Almolda et al., 2011b; Wlodarczyk et al., 2014), support the hypothesis that parenchymal DCs are derived from the differentiation of local cells, probably microglia, on the basis that the expression of some of the surface antigens commonly used for the identification of DCs, such as CD11c, MHCII and CD86, are found in activated microglial cells. In addition, a study using the CD11c-GFP mouse, which expresses the GFP protein under the control of the CD11c promoter, the pan-marker of DCs, has reported the presence of CD11c+ cells not only in the choroid plexuses and perivascular space but also in the juxtavascular parenchyma of non-lesioned CNS (Prodinger et al., 2010). Interestingly, these authors found that almost all CD11c+ cells in the juxtavascular parenchyma presented markers of microglial cells such as Iba1 and CD11b, indicating that, presumably, a subpopulation of microglial cells is able to express DC markers in steady-state conditions. Even more, an interesting study (Anandasabapathy et al., 2011) using the Flt3-treatment, a transcription factor involved in the generation of DCs (Waskow et al., 2008; Kingston et al., 2009), to induce the expansion of DCs in transgenic mice carrying the EYFP fluorescent protein under the control of the CD11c promoter, demonstrated the presence of two different populations of CD11c+ cells within healthy CNS. These two populations corresponded to a population of EYFP+ cells located in the choroid plexuses and meninges whose number increased after Flt3 treatment and another discrete population of EYFP+ cells located in CNS parenchyma with ramified morphology whose number remains stable after the treatment. Flow cytometry studies of these two populations demonstrated that the EYFP+ cells in the choroid plexuses and meninges presented a profile of CD45^high^/MHCII+ DCs, whereas those EYFP+ cells in the parenchyma corresponds to CD45^int^/MHCII- microglial cells (Anandasabapathy et al., 2011). Furthermore, other works (Wlodarczyk et al., 2014) using flow cytometry for different DCs markers have reported the existence of two populations of DCs in EAE-induced animals *in vivo*: CD11c+ DCs and CD11c+ microglia. Interestingly, both populations showed a similar ability to induce T-cell proliferation *in vitro* but, once activated, those T-cells showed a different cytokine profile, suggesting that both populations can play different functions in T-cell activation (Wlodarczyk et al., 2014).

Altogether, these studies indicate, as previously suggested by other authors (Ghosh, 2010), that in addition to professional DCs located in meninges, choroid plexuses and the perivascular space, there is a population of microglial cells that, according to environmental cues, can acquire the phenotype of DCs and consequently may act as professional APCs. One issue to be resolved is if these parenchymal DCs that come from microglia develop the same functions as other DCs or, conversely, if both populations in the CNS have different roles regulating the immune response.

## Concluding Remarks

Current research suggests that the net effect of the acquired immune response within the CNS must depend not only on the number of lymphocytes and APCs, but must also be directly related to the specific subtype of infiltrated lymphocytes, the particular phenotype of the APC in each situation and the specific micro-environment in which the communication between these two cells takes place. Whether the principal intercomunicators in the cross talk with T-cells are microglial cells, professional DCs or both is an intriguing question, still under discussion, and should be subject to thorough investigation. Research to help clarify the question of the origin and a more complete characterization of the phenotype and function of parenchymal DCs in CNS will offer a more comprehensive understanding of the role played by these cells during the evolution of neuroinflammatory processes.

## Conflict of Interest Statement

The authors declare that the research was conducted in the absence of any commercial or financial relationships that could be construed as a potential conflict of interest.
